# Comparative biomechanical evaluation of the U-shaped lumbopelvic stabilization technique in treating unstable sacral fractures

**DOI:** 10.1007/s00402-025-05988-5

**Published:** 2025-07-12

**Authors:** Dennis Nebel, Manuel Ferle, Thorben Schulz, Bastian Welke, Tilman Graulich, Sebastian Decker

**Affiliations:** 1 Laboratory for Biomechanics and Biomaterials, Department of Orthopaedic Surgery, Hannover Medical School, DIAKOVERE Annastift, Anna-von-Borries Str. 1-7, 30625 Hanover, Germany; 2 Department of Ergonomics, Technical University of Munich, 85748 Garching, Munich, Germany; 3 Department of Trauma Surgery, Hannover Medical School, Carl-Neuberg-Str. 1, 30625 Hanover, Germany

**Keywords:** *In vitro* biomechanics, Lumbopelvic stabilization, Sacral fracture, Surgical technique

## Abstract

**Introduction:**

Lumbopelvic fixation is commonly employed to stabilize unstable sacral fractures, particularly U-shaped (US) fractures, which may result in spinopelvic dissociation—leading to significant pain, deformity, and neurological deficits. Due to its superior biomechanical properties, lumbopelvic stabilization (LPS) has become the preferred method for managing such injuries. We aimed to compare the biomechanical stability of US-LPS with conventional LPS and bilateral iliosacral screw (ISS) fixation.

**Materials and methods:**

Six human cadaveric pelvic specimens were subjected to axial (750 N) and torsional (8 Nm) loading using a material testing machine (MTM). Seven configurations of LPS were evaluated. The range of motion (ROM) between three anatomical bony segments—the third lumbar vertebral body (LBV3), the first sacral vertebral body (SVB1), and the Crista Iliaca (CI)—was analyzed using an optical tracking system. Measurements included craniocaudal translation and anterior–posterior tilt under axial loading and internal–external rotation under torsional loading. Stiffness was calculated using force–displacement curves obtained via the MTM’s integrated load cells and displacement transducers.

**Results:**

Both LPS and US-LPS configurations demonstrated reduced ROM and increased stiffness compared to ISS fixation under axial and torsional loading. US-LPS exhibited marginally greater stiffness than standard LPS. The use of additional cross-connectors in both LPS groups had minimal to no measurable impact on ROM or overall stability.

**Conclusions:**

US-LPS offers slightly enhanced biomechanical stability over conventional LPS in the fixation of unstable US sacral fractures. In contrast, standalone ISS fixation did not improve stability compared to the unfixed condition in this cadaveric model.

**Supplementary Information:**

The online version contains supplementary material available at 10.1007/s00402-025-05988-5.

## Introduction

Lumbopelvic fixation is most commonly employed to stabilize unstable sacral fractures in patients with trauma, with U-shaped (US) sacral fractures being particularly prevalent. These fractures, originally defined as bilateral longitudinal fractures combined with a transverse component, result in spinopelvic dissociation [1]. They are often associated with ligamentous injury—particularly in cases of high-energy trauma—and are predisposed to dislocation due to significant biomechanical instability. As a result, long-term consequences frequently include neurological deficits, chronic pain, deformity, and diminished quality of life [1, 2].

Although these fractures were initially characterized in the context of high-energy trauma, analogous osseous fracture patterns have been observed in elderly individualy following minor trauma or spontaneous fractures [3]. In this population, neurological deficits are less common; however, pain-induced immobilization is a predominant clinical concern.

In clinical practice, key therapeutic goals include pain reduction, early mobilization, and the prevention of neurological deterioration. Various surgical techniques have been developed to stabilize the lumbopelvic junction and promote fracture healing. Iliosacral screw (ISS) fixation remains a widely used approach for moderate sacral and pelvic fractures and was among the earliest techniques applied to US fractures [4, 5]. Despite its popularity, the biomechanical efficacy of ISS remains debated, prompting the development of various screw designs to enhance stability. Although several studies have reported favorable outcomes with isolated ISS fixation, heterogeneous patient populations and variable fracture instability make it difficult to draw definitive conclusions [2, 4, 6].

Lumbopelvic stabilization (LPS) has become a standard technique in cases requiring enhanced biomechanical support. This method uses pedicle screw constructs to restore structural integrity at the base of the spine and can be combined with ISS for additional support, a combination being known as triangular stabilization. Numerous modifications of lumbopelvic fixation have been proposed, including variations in construct length and implant configuration [2, 7] to [8]. While LPS generally provides sufficient rigidity to facilitate early mobilization and protect neurologic structures, open posterior surgical approaches carry a risk of soft tissue complications [8]. Recently, percutaneous techniques have emerged to mitigate these issues, enabled by advances in implant technology [9].

In 2019, Decker et al. introduced the US-LPS technique, a novel configuration in which iliac screws are connected bilaterally to stabilize vertical fracture lines, followed by lumbar fixation with pedicle screws [10]. Although promising, the biomechanical performance of this US configuration compared to conventional LPS remains unclear and has not been analyzed so far.

In this work, we aimed to evaluate whether US-LPS offers superior biomechanical stability compared to conventional lumbopelvic fixation, using the same number of fixed segments. Additionally, its performance is compared with that of bilateral ISS fixation.

## Materials and methods

### Ethics approval

was not required for this study, as it involved commercially sourced human anatomical specimens. This was confirmed by written documentation from the local ethics committee dated July 4, 2023.

### Specimen preparation

Six freshly frozen human cadaveric pelvises (Science Care Inc., Phoenix, AZ, USA), including the lumbar spine through to the proximal femur, were used for testing (three male and three female). The donors had a mean age of 63.4 ± 14.5 (range: 48–85) years and a mean body mass index of 25.8 ± 3.8 kg/m². Bone mineral density was assessed via osteodensitometry, yielding a mean T-score of -2.05 ± 1.15 across specimens.

All specimens were stored at -20 °C and thawed at room temperature for 24 h prior to preparation and testing. The proximal femora were stripped of soft tissue and embedded in a cold-curing resin (RenCast FC52/53, Huntsman Advanced Materials, Salt Lake City, UT, USA) to ensure rigid fixation during testing.

### Test setup

Biomechanical testing was conducted using a material testing machine (MTS MiniBionix 858; MTS Systems Corporation, Eden Prairie, MN, USA). The distal femora were embedded in two aluminum cylinders, which were mounted on a base plate affixed to the material testing machine (MTM) and allowed unrestricted mediolateral movement, while anteroposterior translation was constrained. The third lumbar vertebral body (LVB3) was rigidly mounted onto a plate and connected to the upper actuator of the testing system via a Cardan joint, allowing for multiplanar articulation.

**Fig. 1:**
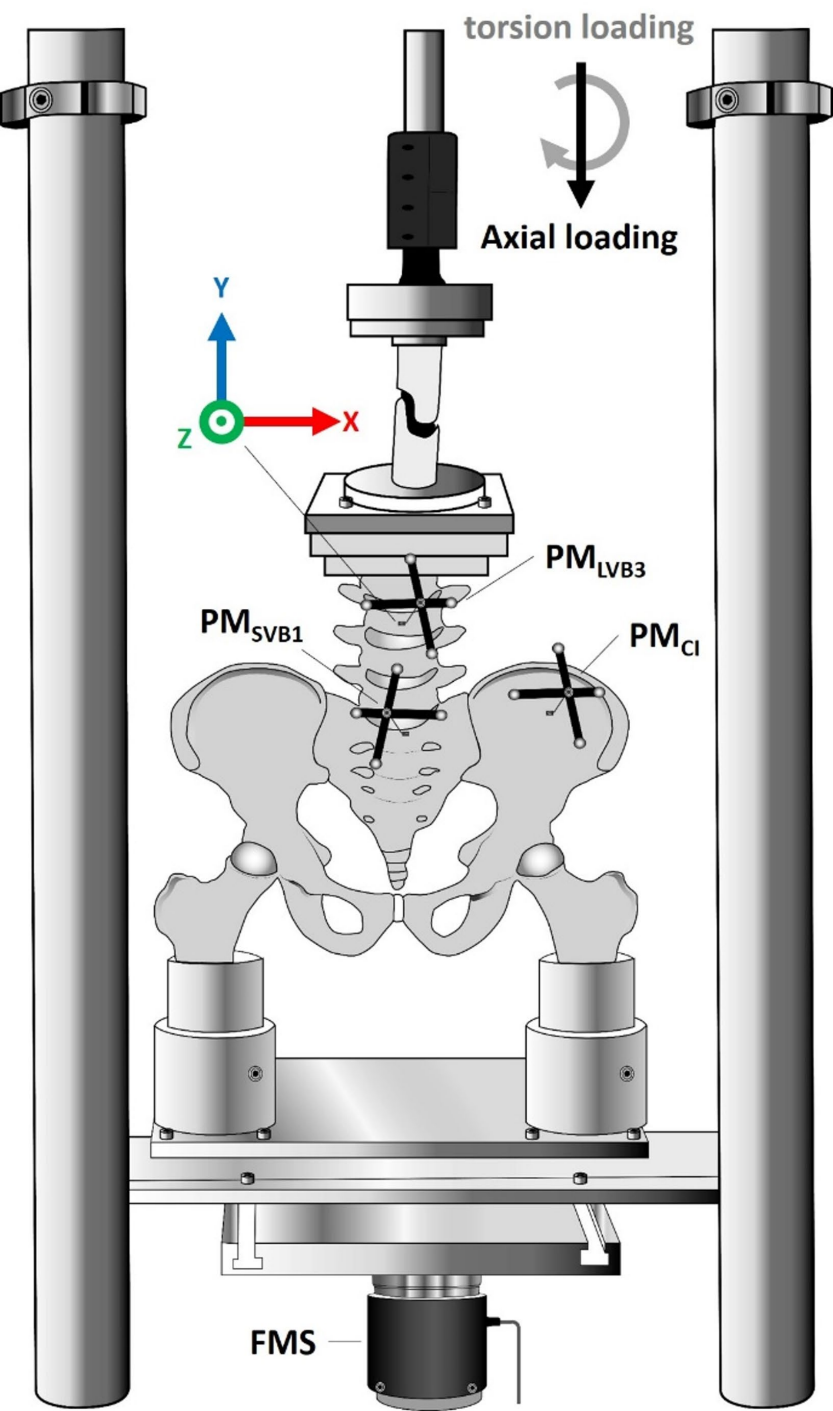
In vitro biomechanical test setup. The pelvis is mounted to the force and moment sensor (FMS) of the material testing machine (MTM) via the two femora, each embedded in aluminum pots. Three passive markers (PM) were affixed to anatomical bony segments (LVB3, SVB1, and CI) using K-wires. The reference coordinate system originates at LVB3. Axial (black) and torsional (grey) loading scenarios were applied via a stamp mounted on a Cardan joint

To assess segmental kinematics, an optical tracking system (NDI Polaris P4, NDI, Waterloo, Canada) was used to record motion between three anatomical bony segments: the LVB3, the first sacral vertebral body (SVB1), and the crista iliaca (CI). Passive reflective markers were affixed to each of these bony segments using Kirschner wires (K-wires). The reference coordinate system for measuring relative motion was established based on LVB3, in accordance with the convention described by Crawford et al. [11]: the x-axis was defined as mediolateral (right to left), the y-axis as craniocaudal (inferior to superior), and the z-axis as anteroposterior (posterior to anterior) (Fig. 1).

### Surgical procedure

Standard polyaxial pedicle screws (6 × 45 mm; Expedium, Johnson & Johnson, New Brunswick, NJ, USA) were bilaterally inserted into the L3 and L4 vertebrae. Additionally, polyaxial iliac screws (8 × 80 mm; Expedium, Johnson & Johnson) were placed bilaterally. The selection of L3 and L4 levels was based on the original percutaneous technique described by Decker et al. [10]. While there is consensus on minimizing the length of lumbopelvic constructs, extending the fixation to L3/L4 may accommodate concurrent L5 fractures and improve overall stability, if clinically necessary. Nevertheless, in the majority of sacral fractures necessitating lumbopelvic fixation, L5 is sufficient as the uppermost instrumented vertebra. However, of course in most sacral fractures requiring lumbopelvic fixation, L5 is enough as upper most instrumented vertebra.

During testing, these screws were connected in various LPS configurations using 5.5 mm titanium rods (Expedium, Johnson & Johnson). For ISS placement, bilateral screw channels were predrilled using 2.8 mm guide wires, and 7.3 mm lag screws (Johnson & Johnson) were subsequently inserted.

Ligamentous structures were preserved as much as possible during soft tissue dissection. To simulate a US sacral fracture, a vertical osteotomy was performed from the sacral ala to the S2 foramen bilaterally, followed by a transverse osteotomy between the S2 foramina. These osteotomies were performed using a small oscillating saw and chisel, with careful preservation of surrounding soft tissues.

### Reconstruction configurations and testing protocol

Each specimen was tested under the following seven sequential conditions and implant configurations:

- Intact specimen.
  - intact → *I*.
- Fractured specimen.
  - without instrumentation → *F*.
  - with lumbopelvic stabilization L3/4-Ilium → *LPS*.
  - with lumbopelvic stabilization L3/4-Ilium and cross connector → *LPS-CC*.
  - with bilateral iliosacral screws → *ISS*.
  - with U-shaped lumbopelvic stabilization → *US-LPS*.
  - with U-shaped lumbopelvic stabilization and cross-connector → *US-LPS-CC*.

**Fig. 2:**
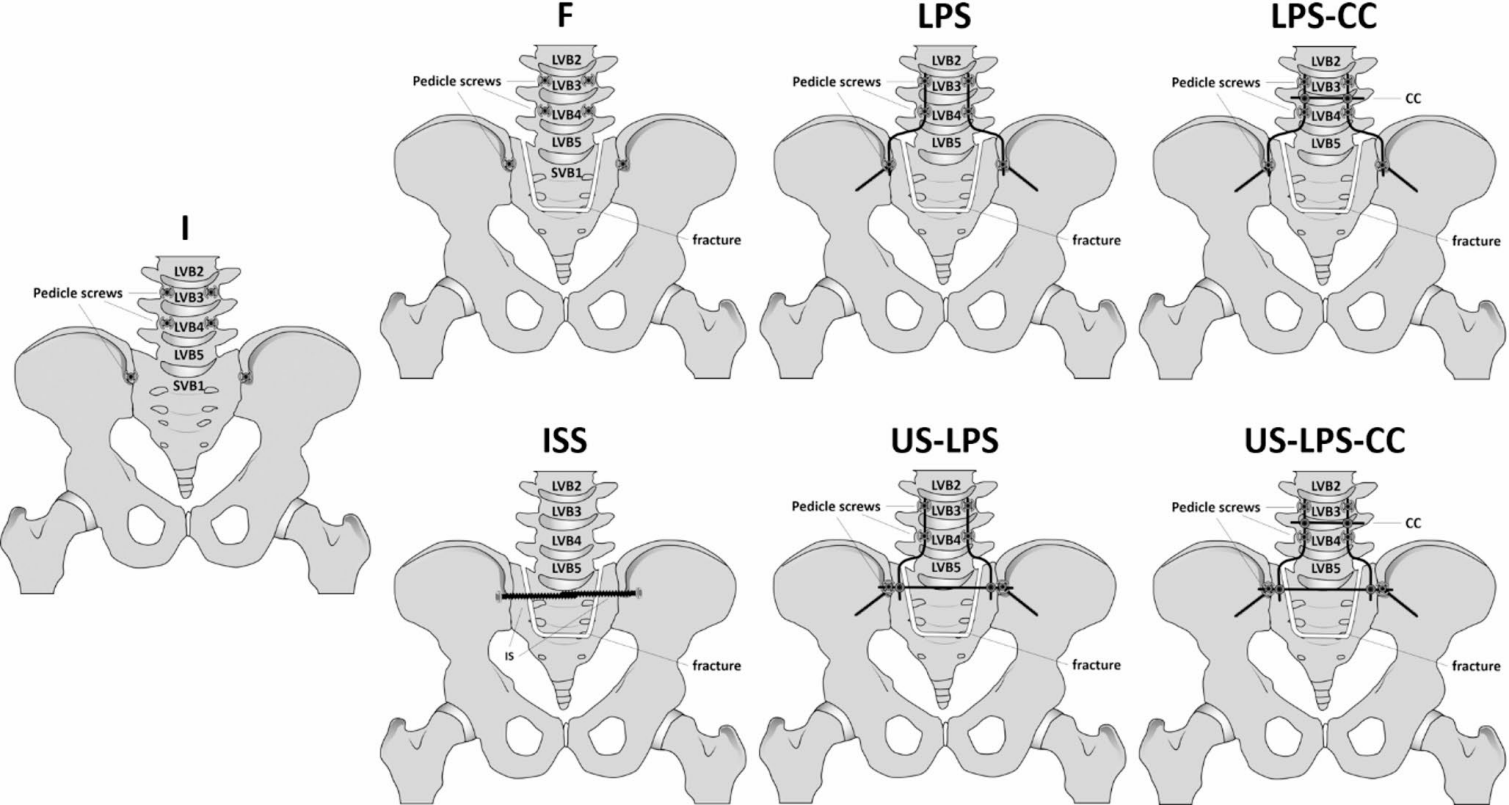
Schematic overview of the seven tested conditions and instrumentation configurations. These include (I) intact specimen, (F) fractured specimen without instrumentation, (LPS) lumbopelvic stabilization L3/4-Ilium, (LPS-CC) LPS with cross-connector, (ISS) bilateral iliosacral screws, (US-LPS) U-shaped lumbopelvic stabilization, and (US-LPS-CC) US-LPS with cross-connector

These configurations and test stages are illustrated in Fig. 2.

The testing protocol involved five loading cycles with an initial preload of 100 N, applied under two loading conditions: axial compression (750 N) and torsional loading (8 Nm), as previously described [12, 13]. The loading velocities were set at 0.5 mm/s for axial loading and 1°/s for torsional loading. Throughout the testing, specimens were regularly moistened with 0.9% saline solution to prevent tissue desiccation.

Only data from the fifth loading cycle were analyzed. During axial loading, the range of motion (ROM) was assessed in the craniocaudal direction (Y-axis) and as rotation around the transverse axis (C). Under torsional loading, ROM was measured as rotation around the longitudinal axis (B). Additionally, stiffness for each construct was calculated during the fifth cycle. Stiffness was defined as the slope of a linear regression fit to the force–displacement curve, which was obtained from the MTM’s built-in displacement transducers and load cells.

### Statistical analysis

One specimen was excluded due to severe damage sustained during testing. Muscles and extraneous soft tissues were dissected from all specimens, including from the LVB3. Inferential statistical analysis was not performed due to the small sample size (*n* = 5) and occasional incomplete datasets. Data loss was attributed to the failure to capture all five loading cycles, likely caused by optical marker tracking errors.

As a result, only descriptive statistics are reported. Data are presented as median values with corresponding 95% confidence intervals (CIs). Additionally, results are visualized using boxplots overlaid with scatterplots to display individual data points.

## Results

###  Axial loading—cranial–caudal translation

#### SVB to LVB

**Fig. 3:**
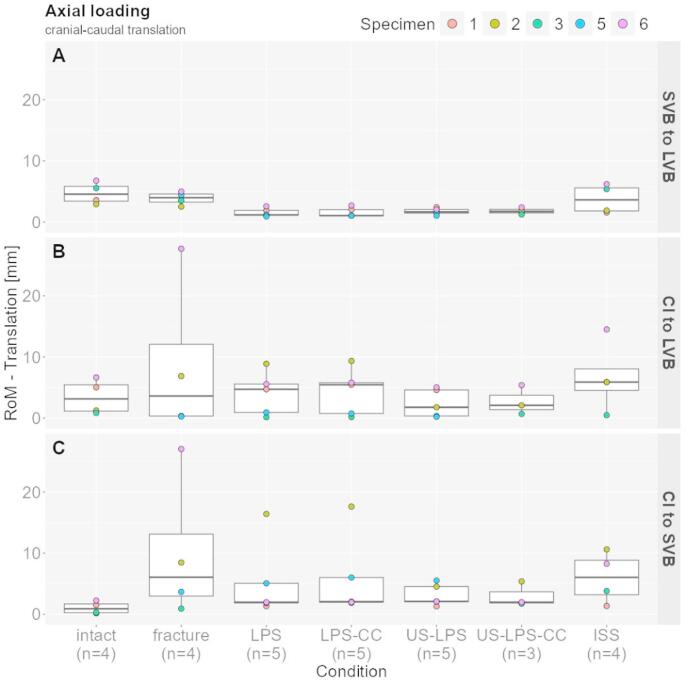
Range of motion under axial loading (cranial–caudal translation). Box plots with scatter points display translational range of motion for three segmental comparisons: (A) SVB to LVB, (B) CI to LVB, and (C) CI to SVB

The intact condition showed a median ROM of 4.56 mm (95% CI: 2.92–6.75), and the fractured condition had a 13% lower ROM (3.99 mm, 95% CI: 2.52–4.99). The LPS method reduced the ROM by 71% (1.17 mm, 95% CI: 0.92–2.58) compared with the fractured condition. The additional CC reduced the ROM by 73% (1.06 mm, 95% CI: 1.04–2.71). The US-LPS method reduced the ROM by 59% (1.65 mm, 95% CI: 1.04–2.43) and 56% (1.74 mm, 95% CI: 1.25–2.40), with an additional CC use. ISS enabled a 9% reduction in the ROM (3.63 mm, 95% CI: 1.57–6.19) (Fig. 3A).

#### CI to LVB

The ROM of the intact condition was 3.15 mm (95% CI: 0.85–6.65), which was 13% lower than the fracture condition (3.62 mm, 95% CI: 0.28–27.69). The standard LPS procedure increased the ROM by 30% (4.72 mm, 95% CI: 0.16–8.89) and 51% (5.46 mm, 95% CI: 0.17–9.35) with an additional CC use. In contrast, the US-LPS application reduced the ROM by 51% (1.78 mm, 95% CI: 0.23–5.05) and 42% (2.10 mm, 95% CI: 0.69–5.40) with a CC. The greatest movement was observed with the ISS, increasing the ROM by 63% (5.90 mm, 95% CI: 0.48–14.50) (Fig. 3B).

#### CI to SVB

The largest ROM was seen in the fractured state (6.06 mm, 95% CI: 0.92–27.02), whereas the smallest ROM was in the intact state, which was 85% lower (0.90 mm, 95% CI: 0.15–2.25). An approximately identical reduction in ROM between 65% and 67% was observed in all LPS treatments (LPS: 1.97 mm, 95% CI: 1.32–16.39; LPS-CC: 2.06 mm, 95% CI: 1.86–17.61; US-LPS: 2.10 mm, 95% CI: 1.30–5.51; US-LPS-CC: 1.97 mm, 95% CI: 1.74–5.37). ISS use did not show any differences compared to the fractured state (6.03 mm, 95% CI: 1.36–10.60) (Fig. 3C).

### Axial loading—anterior–posterior tilting (in sagittal plane)

#### LVB to SVB

**Fig. 4:**
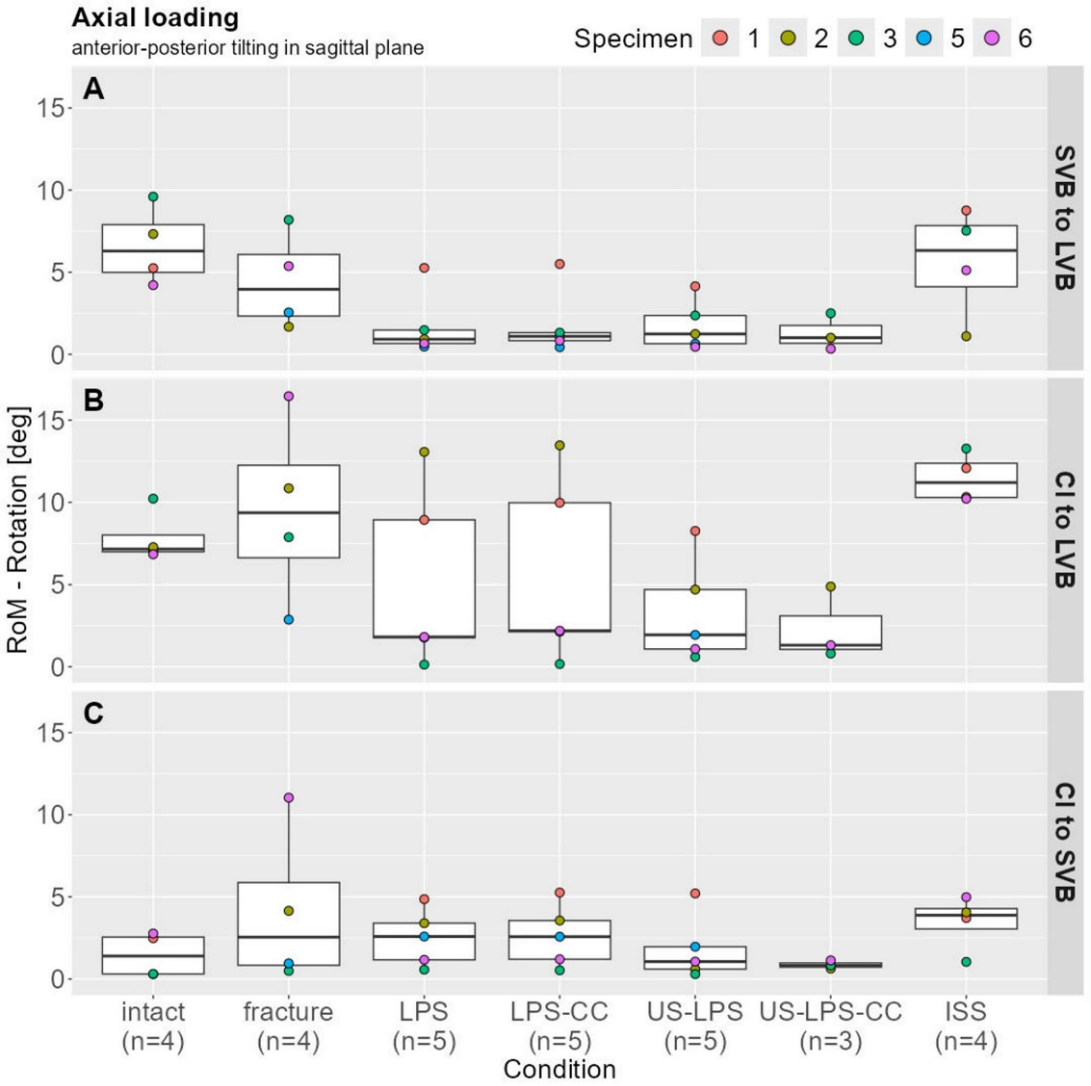
Range of motion under axial loading (anterior–posterior tilting in the sagittal plane). Box plots with scatter points depict angular motion for the following kinematic segments: (A) SVB to LVB, (B) CI to LVB, and (C) CI to SVB

The fractured condition was approximately 37% less than the intact condition (3.96 mm, 95% CI: 1.68–8.19 vs. 6.29 mm, 95% CI: 4.22–9.60). The LPS and the US-LPS state reduced the ROM by 77% (0.92 mm, 95% CI: 0.47–5.26) and 69% (1.24 mm, 95% CI: 0.46–4.15) compared with the fractured state. No additional reduction in the ROM was observed with the connector in either LPS instrumentation. Therefore, the ROM was similarly reduced at 72% for LPS-CC (1.10 mm, 95% CI: 0.44–5.50) and 74% for US-LPS-CC (1.01 mm, 95% CI: 0.34–2.50) compared with the fractured condition. The ISS ROM was comparable to that of the intact state (6.32 mm, 95% CI: 1.10–8.76) (Fig. 4A).

#### CI to LVB

In the intact state, the median ROM was 7.15 mm (95% CI: 6.84–10.22), whereas, in the fractured state, the ROM increased by approximately 30% (9.36 mm, 95% CI: 2.87–16.46). LPS application resulted in a significant ROM reduction of 81% (1.82 mm, 95% CI: 0.14–13.07) or 77% (2.20 mm, 95% CI: 0.17–13.47) when accompanied by an additional connector, in comparison to the fractured condition. Comparable ROM reductions of 79% and 86% were observed for US-LPS with and without a connector (1.95 mm, 95% CI: 0.61–8.25 vs. 1.32 mm, 95% CI: 0.81–4.88), respectively. The ISS ROM condition had a median value of 11.20 mm (95% CI: 10.22–13.27), indicating a 120% increase compared to the fractured condition (Fig. 4B).

#### CI to SVB

The fractured condition had an 82% (2.55 mm, 95% CI: 0.50–11.04) increase in ROM compared with the intact condition (1.40 mm, 95% CI: 0.30–2.77). No reduction in the ROM was observed for the LPS treatment with and without the additional connector compared with the fracture condition (2.59 mm, 95% CI: 0.57–4.86 vs. 2.58 mm, 95% CI: 0.53–5.26). Conversely, the US-LPS treatment with and without the connector resulted in a ROM reduction of 59% (1.06 mm, 95% CI: 0.30–5.21) and 68% (0.82 mm, 95% CI: 0.63–1.13). A 52% increase in the ROM was observed in the ISS condition compared with the fractured state (3.89 mm, 95% CI: 1.04–4.98) (Fig. 4C).

### Torsional loading—internal–external rotation (in transverse plane)

#### LVB to SVB

**Fig. 5:**
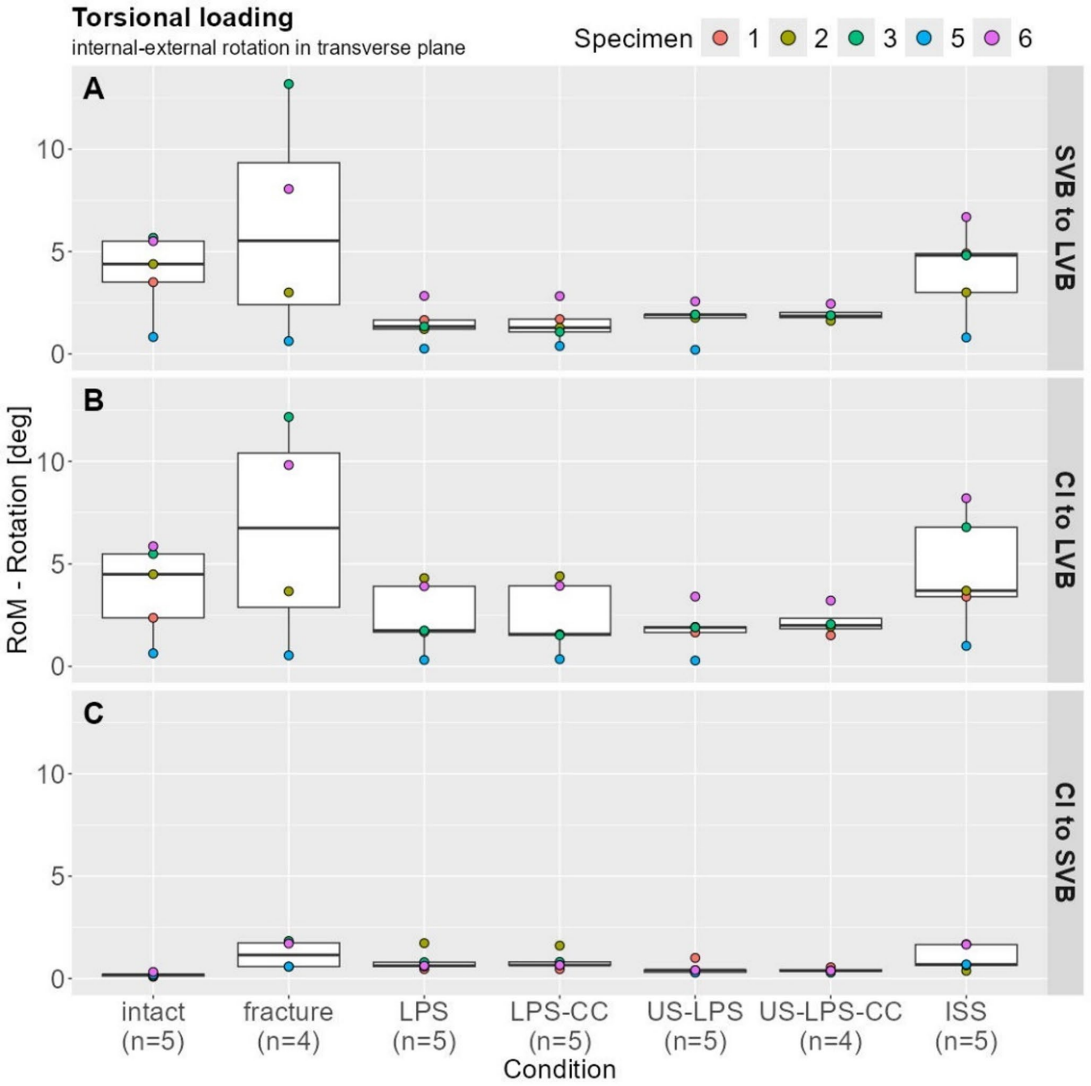
Range of motion under torsional loading (internal–external rotation in the transverse plane). Box plots with scatter points show torsional rotation for (A) SVB to LVB, (B) CI to LVB, and (C) CI to SVB

The median ROM in the intact state was 4.39 mm (95% CI: 0.83–5.67), which increased by approximately 26% (5.53 mm, 95% CI: 0.63–13.18) in the fractured state. The LPS and the US-LPS treatment, with and without connectors, respectively, reduced the fractured state ROM by 65–77% (LPS: 1.34 mm, 95% CI: 0.26–2.83; LPS-CC: 1.28 mm, 95% CI: 0.39–2.82; US-LPS: 1.92 mm, 95% CI: 0.20–2.56; US-LPS-CC: 1.86 mm, 95% CI; 1.62–2.45). ISS reduced the ROM by approximately 12% (4.81 mm, 95% CI: 0.80–6.69) (Fig. 5A).

#### CI to LVB

The intact condition ROM was 4.50 mm (95% CI: 0.64–5.85), which was 33% lower than the fracture condition (6.74 mm, 95% CI: 0.54–12.17). The fractured state ROM was reduced by 70–77% with all LPS treatments (LPS: 1.75 mm, 95% CI: 0.31–4.31; LPS-CC: 1.57 mm, 95% CI: 0.35–4.40; US-LPS: 1.90 mm, 95% CI: 0.29–3.41; US-LPS-CC: 2.00 mm, 95% CI: 1.52–3.21). The ISS was reduced by 45% compared with the fractured condition (3.70 mm, 95% CI: 1.00–8.20) (Fig. 5B).

#### CI to SVB

The largest ROM was observed in the fractured state (1.15 mm, 95% CI: 0.58–1.84), whereas the smallest ROM was in the intact state, which was 84% lower (0.19 mm, 95% CI: 0.09–0.33). LPS and LPS-CC treatment reduced the ROM in the fractured state by 45% (0.63 mm, 95% CI: 0.46–1.73) and 43% (0.66 mm, 95% CI: 0.46–1.61). Treatment with US-LPS and US-LPS-CC reduced ROM by 64% (0.41 mm, 95% CI: 0.29–1.01) and 66% (0.39 mm, 95% CI: 0.29–0.55), compared with the fractured condition. In the fractured state, the ROM was reduced by 40% with ISS fixation, resulting in a median value of 0.69 mm (95% CI: 0.38–1.68) (Fig. 5C).

### MTM

#### Axial

**Fig. 6:**
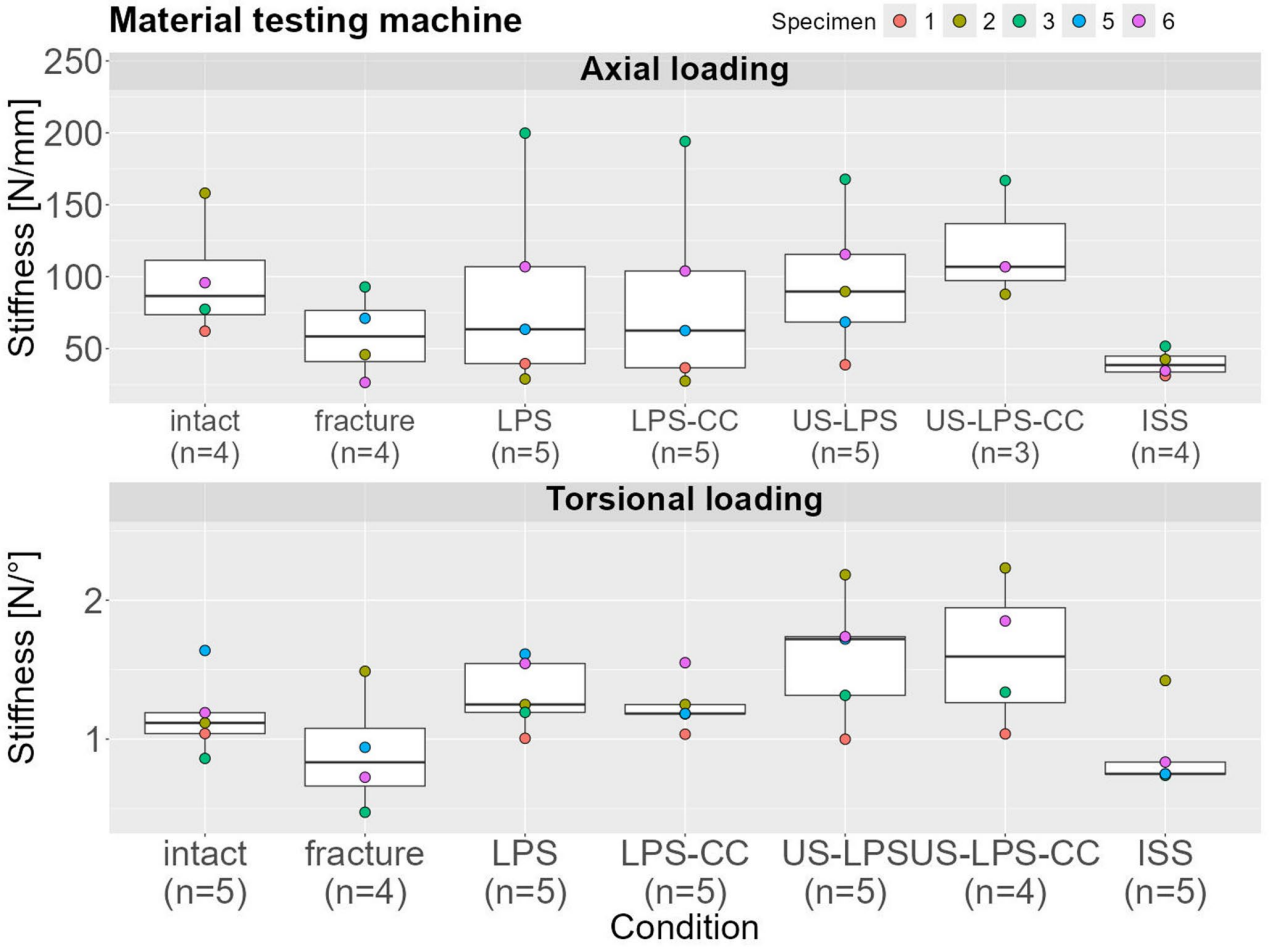
Construct stiffness under axial and torsional loading. Box plots represent stiffness values derived from the slope of the linear portion of the load-displacement curve in the fifth cycle under axial loading and torsional loading. Data were obtained using displacement transducers and load cells integrated into the MTM

The median stiffness was 86.62 mm (95% CI: 62.15–158.09) under axial loading in the intact state. In comparison, the stiffness in the fractured state was 33% lower (58.46 mm, 95% CI: 26.49–92.89). The stiffness was reduced with ISS by 59% (38.58 mm, 95% CI: 31.17–51.67). The stiffness of the treatment with LPS (63.44 mm, 95% CI: 28.94–199.82) and LPS-CC (62.56 mm, 95% CI: 27.44–194.08) slightly increased compared to the fractured condition. The US-LPS (89.69 mm, 95% CI: 38.77–167.73) and US-LPS-CC (106.89 mm, 95% CI: 87.79–166.88) treatments showed higher stiffness than that of the intact condition by 4% and 23%, respectively (Fig. 6).

#### Torsion

The median stiffness in the intact state was 1.12 mm (95% CI: 0.86–1.64) under torsional loading, whereas, in the fractured state, the stiffness was 26% lower (0.83 mm, 95% CI: 0.47–1.49). The stiffness was reduced with ISS by 33% (0.75 mm, 95% CI: 0.74–1.42). The stiffness of the treatment with LPS, LPS-CC, US-LPS, and US-LPS caused stiffness increases between 5% and 54%: LPS (1.25 mm, 95% CI: 1.01–1.61); LPS-CC (1.18 mm, 95% CI: 1.04–1.55); US-LPS (1.72 mm, 95% CI: 1.00–2.18); US-LPS-CC (1.59 mm, 95% CI: 1.04–2.23) (Fig. 6).

## Discussion

In this work, we aimed to evaluate the biomechanical stability provided by various LPS techniques compared to bilateral ISS fixation in the treatment of US sacral fractures. Under axial loading, all forms of LPS significantly reduced the translational ROM across all measured segmental kinematics compared to the non-instrumented fractured state. Specifically, cranial–caudal translation was effectively limited in all lumbopelvic constructs.

No relevant differences were observed between the LPS and LPS-CC or between the US-LPS and US-LPS-CC groups. However, ISS fixation resulted in comparatively less rigid stabilization, as illustrated in Fig. 3. Similar outcomes were found when analyzing anterior–posterior tilting in the sagittal plane under axial loading. All LPS forms effectively reduced rotational ROM, which was not achieved with ISS alone. Although the differences among lumbopelvic constructs were modest, the US-LPS and US-LPS-CC conditions demonstrated the least variability, particularly in comparisons between the CI and both the LVB3 and SVB1, as shown in Figs. 3 and 4. US-LPS showed marginally superior stability compared to LPS and LPS-CC.

Under torsional loading, ISS fixation had no appreciable effect on stabilization, while all forms of lumbopelvic instrumentation sufficiently stabilized the fracture. Notably, rotational movement between CI and LVB was most effectively restricted by US-LPS and US-LPS-CC.

These findings suggest that US-LPS, with or without a cross-connector, provides marginally greater biomechanical stability compared to conventional LPS. In contrast, ISS alone was not biomechanically beneficial in our cadaveric fracture model. The enhanced rigidity of US-LPS may offer a valuable alternative for the fixation of complex spinopelvic injuries [10].

It is important to note that due to the limited sample size (*n* = 5), statistical significance could not be evaluated. Consequently, only descriptive statistics were reported. Despite this limitation, biomechanical trends could be clearly demonstrated (Figs. 3, 4 and 5).

ISS is primarily designed to provide compression across vertical fracture lines. However, these screws do not neutralize shear forces, which may explain the poor biomechanical performance observed in this study [6, 14]. This limitation has led to the development and recommendation of lumbopelvic instrumentation by various authors [15].

Surgeons are encouraged to adopt minimally invasive surgical techniques where possible. Modern implant systems have facilitated such approaches, allowing for reduced soft tissue disruption. While LPS constructs anchored at L5 are commonly used and provide enough stability most times, there might be indications for more lumbar anchorage. In our study, we compared US-LPS (which includes a cross-connection between the iliac screws) to standard LPS to assess whether the added complexity of this minimally invasive construct results in improved biomechanical performance. Our results suggest a slight biomechanical advantage for US-LPS.

Treatment of complex sacral fractures remains challenging, and implant configurations must be tailored to the individual case to optimize fixation strength while avoiding overtreatment [2, 7] to [8, 10, 16]. It is essential to distinguish between fracture types; for example, a geriatric US sacral fracture with intact ligaments and minimal displacement differs substantially in biomechanical demands from a high-energy traumatic fracture with complete ligament disruption. Therefore, implant choice should be guided by fracture pattern, patient characteristics, and biomechanical requirements.

Based on the results of this study and our clinical experience, we advise against the standalone use of ISS in the treatment of unstable US sacral fractures. Lumbopelvic or triangular stabilization offers superior biomechanical performance in such cases [15]. Specifically, US-LPS and US-LPS-CC appear to be reliable configurations for achieving maximum stability. However, given that these techniques typically require fixation of more than one lumbar segment, their use should be reserved for cases involving complex trauma and/or severe biomechanical instability, i.e. when L5 is not appropriate as upper-most instrumented vertebra during lumbopelvic fixation.

In contrast, ISS fixation may still be appropriate in elderly patients with insufficiency fractures where ligamentous structures remain intact. The poor ISS performance in our study may be attributable to the extent of instability introduced by the osteotomy model, as well as reduced bone quality in the SVB1.

Nevertheless, lumbopelvic fixation is not without drawbacks. It is associated with significant complication and reoperation rates [17]. Minimally invasive techniques can reduce infection rates and soft tissue damage but should still be applied judiciously. While increased construct length or the number of implants generally enhances biomechanical stability, this may not always be necessary and should be weighed against the risk of complications and overtreatment [18].

This study had several limitations. It was an in vitro biomechanical investigation using human cadaveric specimens in a simulated immediate postoperative state. Thus, factors such as biological healing, soft tissue recovery, prosthesis integration, and muscle function could not be assessed. Long-term stability was not tested. Additionally, the sample size was small (*n* = 5), and some data were incomplete due to marker tracking issues, precluding inferential statistical analysis. The use of the NDI Polaris P4 passive optical navigation system caused some problems. Accurate kinematic tracking necessitated the visibility of all four reflective markers on a given segment to the camera, a condition often compromised by marker occlusion (e.g., one marker blocked by another marker or markerset, ghost reflections (e.g., from moist tissue surfaces or shiny surfaces), and angular limitations (e.g., marker tilt exceeding 45–60° during movements). These factors led to intermittent tracking failures and thus loss of kinematic data, especially during rotation in cyclic loading.

This study compared the biomechanical rigidity of different LPS techniques. Our findings support the use of US-LPS and US-LPS-CC in the treatment of unstable US sacral fractures compared to conventional lumbopelvic stabilization L3/4-Ilium while the classic construct length L5-Ilium was not part of this study. We strongly advise against the isolated use of ISS in these injuries. However, in geriatric insufficiency fractures, ISS may be sufficient. It is important to note that the osteotomy model used in this study more closely resembles unstable, high-energy trauma fractures than geriatric insufficiency fractures.

## Conclusions

US-LPS was slightly more stable than conventional LPS techniques for fixing unstable US sacral fractures in our osteotomy model. Only ISS did not increase biomechanical stability compared to no fixation in our cadaver model, which is in contrast to other studies. While our sample size was small, interpretation of results should be done carefully.

## Electronic supplementary material

Below is the link to the electronic supplementary material.

Supplementary Material 1

Supplementary Material 2

## Data Availability

No datasets were generated or analysed during the current study.

## Abbreviations

US: U-shaped
LPS: Lumbopelvic stabilization
ISS: Bilateral iliosacral screw
MTM: Material testing machine
LVB3: Third lumbar vertebral body
SVB1: First sacral vertebral body
CI: Crista Iliaca
K-wires: Kirschner wires
FMS: Force and moment sensor
PM: Passive markers
ROM: Range of motion
CIs: Confidence intervals
